# Hyperammonemic Encephalopathy Secondary to Urinary Tract Infection

**DOI:** 10.7759/cureus.31754

**Published:** 2022-11-21

**Authors:** Asher Gorantla, Anandita Kishore, Areeba Zaman, Michael Ramirez, Harshavardhan Taluru, Nisha Horton, Shruthi Sivakumar, Patrick Geraghty, Samy I McFarlane

**Affiliations:** 1 Internal Medicine, State University of New York Downstate Health Science Center, Brooklyn, USA; 2 Internal Medicine, Sisters of Charity Hospital, Buffalo, USA; 3 Internal Medicine, State University of New York Downstate Health Sciences University, Brooklyn, USA; 4 Neurology, State University of New York Downstate Health Sciences University, Brooklyn, USA

**Keywords:** ammonia, encephalopathy, urease-producing bacteria, urinary tract infection, hyperammonemia

## Abstract

Hyperammonemic encephalopathy (HE) refers to a clinical condition characterized by abrupt alteration in mental status (AMS) with markedly elevated plasma ammonia levels and frequently results in intractable coma and death. While hepatic cirrhosis is by far the most common etiology for hyperammonemia together with drugs such as valproic acid as well as urea cycle disorders, non-hepatic causes of hyperammonemia are rare and pose a clinical challenge. In this report, we describe a case of HE caused by obstructive urinary tract infection due to urease-producing bacteria in a 69-year-old man with two episodes of obstructive uropathy associated with AMS resolving with treatment with antibiotics and lactulose with normalization of ammonia level. We also provide a review of the literature with emphasis on the recognition of this serious entity of HE in the setting of obstructive uropathy to avoid the possible complications that include intractable coma and high mortality from this potentially treatable disorder.

## Introduction

Most commonly, hyperammonemia in adults is associated with the development of cirrhotic liver disease [1-3] and is associated with a poor prognosis [4]. Non-hepatic causes of hyperammonemia are rare and pose a clinical challenge. One such cause is urinary tract infections (UTI) caused by urea-splitting organisms [1]. We aim to describe two episodes of hyperammonemic encephalopathy (HE) in a patient with a UTI without evidence of liver disease.

## Case presentation

We present a 69-year-old man with benign prostatic hyperplasia, pituitary xanthogranuloma status post resection in 2009 and 2011, and hypothyroidism who was transferred from a nursing home for altered mental status. On presentation, his vitals were stable. He was unresponsive but able to protect his airway. He was found to have acute urinary retention (AUR) with approximately 1000ml of urine. A Foley catheter was placed, and labs showed an increased ammonia level of 279 µ/dL, creatinine 1.54 mg/dL (baseline 0.7 mg/dl), and urinalysis was positive for leukocyte esterase. Urine culture grew extended-spectrum beta-lactamase (ESBL) Proteus mirabilis. The patient received parenteral antibiotic therapy and continuous urinary catheter drainage as well as lactulose for hyperammonemia with significant improvement in mental status.

The patient was admitted with a similar presentation seven months earlier for unresponsiveness. During that presentation, the patient had AUR, and the placement of the Foley catheter was difficult due to urethral stricture, requiring assistance from urology. Labs were significant for UTI and an ammonia level of 435 µ/dL. The patient underwent extensive work for hyperammonemia with gastroenterology consultation. The patient’s liver enzymes, abdominal ultrasound along with INR and albumin, both markers of hepatic synthetic function, were within normal range, hence hepatic etiology of elevated ammonia was ruled out. MRI of the brain did not show acute intracranial pathology, the EEG showed diffuse slowing but no seizures, and the lumbar puncture (LP) did not indicate central nervous system infection. Other causes for hyperammonemia, such as elevated valproate level and urea cycle disorders including ornithine transcarbamylase deficiency, were also ruled out. Urine cultures grew ESBL Escherichia coli and urease-positive, coagulase-negative Staphylococcus. The patient was started on lactulose and parenteral antibiotics with improvement in ammonia level to 21 µ/dL, kidney function, and return of mental status back to baseline.

The patient had similar presentations in both admissions. After extensive workup ruling out the other causes, the patient’s abrupt change in mental status was most likely due to HE from obstructive UTI caused by urease-producing bacteria Staphylococcus and Proteus mirabilis.

## Discussion

Here, we report a case of HE caused by obstructive urinary tract infection due to urease-producing bacteria Proteus mirabilis and coagulase-negative Staphylococcus, in the absence of obvious liver disease. Hyperammonemia has varied etiologies [3] which can be further divided into congenital or acquired disorders [5]. Congenital hyperammonemia most often presents in infants but there are rare instances in which these disorders present in late childhood or even adulthood, owing to heterozygous expression of the involved genes. These include urea cycle disorders, especially ornithine transcarbamylase deficiency, N-acetyl glutamate synthase deficiency, and carbamoyl phosphate synthetase 1 deficiency. Methylmalonic acidemia is the most common among organic acidemias. Other rare causes include congenital lactic acidosis conditions, dibasic amino aciduria conditions, and inborn errors of mitochondrial fatty acid oxidation [5]. Acquired hyperammonemia is seen in adults. The following table contains a list of common causes (Table 1).

**Table 1:** Common causes of acquired hyperammonemias

Acquired Hyperammonemias	
Liver failure	The urea cycle is impaired in damaged hepatocytes, leading to the accumulation of nitrogenous wastes in the blood which can rise to toxic levels. Shunting of blood away from the portal circulation or from the portal vein to collateral circulation can also lead to hepatic encephalopathy.
Kidney failure	Inability to excrete excess ammonia as urea
Severe dehydration	
Small intestinal bacterial overgrowth	Urease-producing organisms in the gut that produce excess ammonia
Medications	Valproic acid, carbamazepine, sulfadiazine, ribavirin, salicylates, glycine, etc
Urinary tract infection caused by urease-producing organisms	Proteus, H. pylori, Ureaplasma, Nocardia, Klebsiella, S. epidermidis, S. saprophyticus, Cryptococcus
Reye’s syndrome	

Pathogenesis

**Figure 1:**
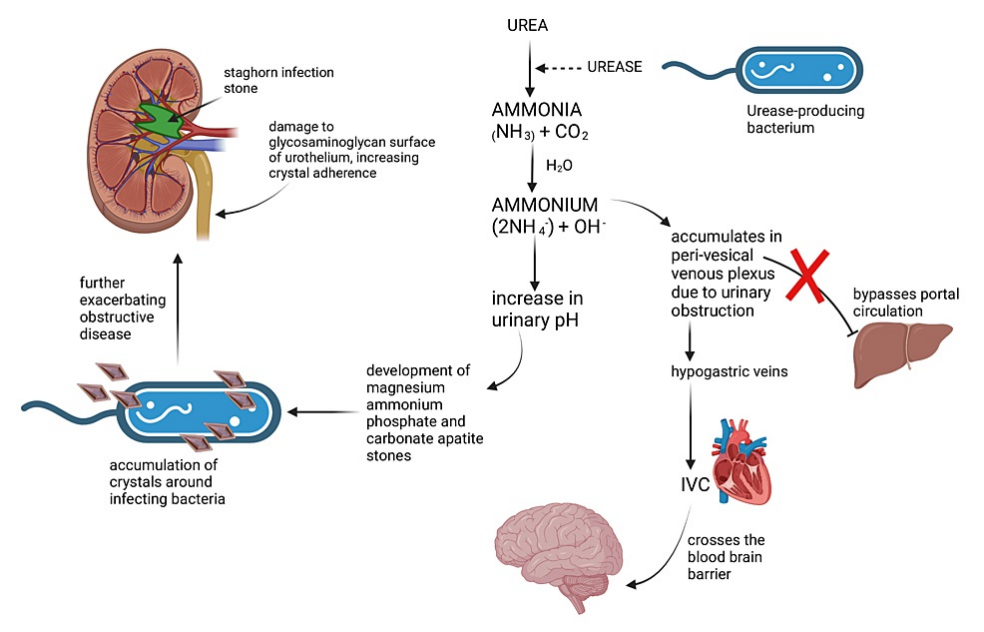
Pathogenesis of acquired hyperammonemia IVC: inferior vena cava This is an original image created by the authors. No further permission for use is required.

Urease-producing bacteria can colonize and survive in the urinary tract (see Figure 1 below for reference). Urease-producing bacteria hydrolyze urea into ammonium ions using urease. The presence of ammonium results in a rise in the urinary pH. Ammonium ions are converted into ammonia in this alkaline environment. In obstructive urinary tract infections, whether from intrinsic or extrinsic blockage, stagnation of urine allows for the transfer of ammonium to the vesical venous plexus and peri-vesical circulation. From here, most venous blood bypasses the portal circulation and liver and instead drains into the hypogastric veins and inferior vena cava. In the brain, elevated serum ammonia levels cross the blood-brain barrier (Figure 1). Astrocytes produce glutamine to counteract ammonia toxicity, leading to increased osmolarity and swelling. In addition, interleukin 1, interleukin 6, tumor necrosis factor, and interferons are released by astrocytes, leading to further damage. The reduction in glutamate receptor expression in astrocytes leads to excessive accumulation of glutamate. These factors collectively damage astrocytes, can lead to the development of flapping tremors (asterixis), and seizures, and can also increase intracranial pressure from cerebral edema which can result in coma and life-threatening cerebral herniation [6].

Treatment

Identifying the underlying cause and prompt treatment is imperative to prevent central nervous system morbidity and possible mortality. Management involves treating the underlying cause along with using medications that lower the plasma ammonia levels such as lactulose, rifaximin, and neomycin. Lactulose acts by facilitating the conversion of NH3 to NH4+. In the gut, bacteria degrade lactulose to create an acidic pH which ultimately reduces blood ammonia levels. In addition, it also has an osmotic effect, promoting peristalsis in the colon due to colonic distension. Rifaximin and neomycin are antibiotics that act on gastrointestinal flora that produce excess ammonia. In patients who develop cerebral edema, hypertonic saline is used to manage the resultant increased intracranial pressure [7]. The use of hyperosmolar agents and propofol for sedation are effective for managing cerebral associated with HE.

## Conclusions

Severe hyperammonemia has high mortality and morbidity even in the absence of liver disease. Appropriate and timely management requires a clear understanding of the fundamental pathophysiology, differential diagnosis, and treatment approaches available. Hyperammonemia secondary to a urinary tract infection with urea-splitting bacteria is rare but is associated with life-threatening sepsis-associated encephalopathy. Hence, it is important to measure and treat the blood ammonia levels in patients with obstructive UTI with an altered level of consciousness.
